# Assessment of leg muscle morphology by contrast-enhanced magnetic resonance imaging (CE-MRI) in patients with peripheral arterial disease

**DOI:** 10.1186/1532-429X-15-S1-P235

**Published:** 2013-01-30

**Authors:** Gerd Brunner, Jean Bismuth, Vijay Nambi, Christie M Ballantyne, William A Zoghbi, Alan Lumsden, Joel Morrisett, Dipan J Shah

**Affiliations:** 1 Baylor College of Medicine, Houston, TX, USA; 2 The Methodist DeBakey Heart & Vascular Center, The Methodist Hospital, Houston, TX, USA

## Background

Peripheral arterial disease (PAD) is associated with functional impairment in the lower extremities (LE). PAD related skeletal muscle loss is a complex and poorly understood process which has been associated with denervation and a decrease in type II muscle fiber mass. Previous studies suggest a potential link between change in muscle morphology and PAD. We hypothesize that imaging can be used to quantify differences in skeletal muscle morphology between PAD patients and controls. We utilized CE-MRI and machine learning using texture features to quantify muscle morphology in PAD patients and healthy controls.

## Methods

Thirty-four individuals (20 PAD patients [median age=67 years, median ankle brachial index=0.62], 14 healthy controls [median age=35 years]) underwent CE-MRI of the LE using a 36-element bilateral LE coil with a 3.0T MRI system. Imaging was performed post reactive hyperemia induced with a bilateral blood-pressure cuff positioned above the knee. Cross-sectional images were acquired at the mid-calf level using a high resolution saturation recovery gradient echo pulse sequence (temporal resolution=409.57ms; slice thickness=10mm; echo time=1.23ms; in plane resolution=1.3x1.3mm). The anterior-, lateral-, and deep posterior muscle compartments and the soleus-, and gastrocnemius muscles were semi-automatically segmented with in-house developed software. The segmented muscle groups were utilized to automatically extract texture features that characterize muscle morphology in the CE-MRI data: 1) texture maps, 2) multi-directional Haralick gray level co-occurrence matrix features; 3) Gabor wavelet features; 4) scale-invariant feature transform features; and 5) intensity histogram feature. Subsequently, a support vector machine (SVM) based framework was developed to classify skeletal muscle tissue as ‘normal' or ‘PAD' by using the extracted features.

## Results

All PAD patients were managed with optimal medical therapy and 10 also underwent LE revascularization. The analysis was performed on the pre-intervention CE-MRI scans of the more symptomatic leg (Figure). The 2-class SVM was trained using a histogram intersection kernel with a cost factor=1,100. The SVM performance was optimized using feature selection in a randomly selected training dataset. Using cross-validation, the SVM correctly identified muscle tissue of PAD patients with sensitivity, specificity, positive predictive value, negative predictive value, and overall classification accuracy of 90.0%, 85.71%, 90.0%, 85.71%, and 88.24%, respectively. A total of 2 patients and 2 controls were incorrectly identified (false positives and false negatives) out of all 34 individuals.

**Figure 1:**
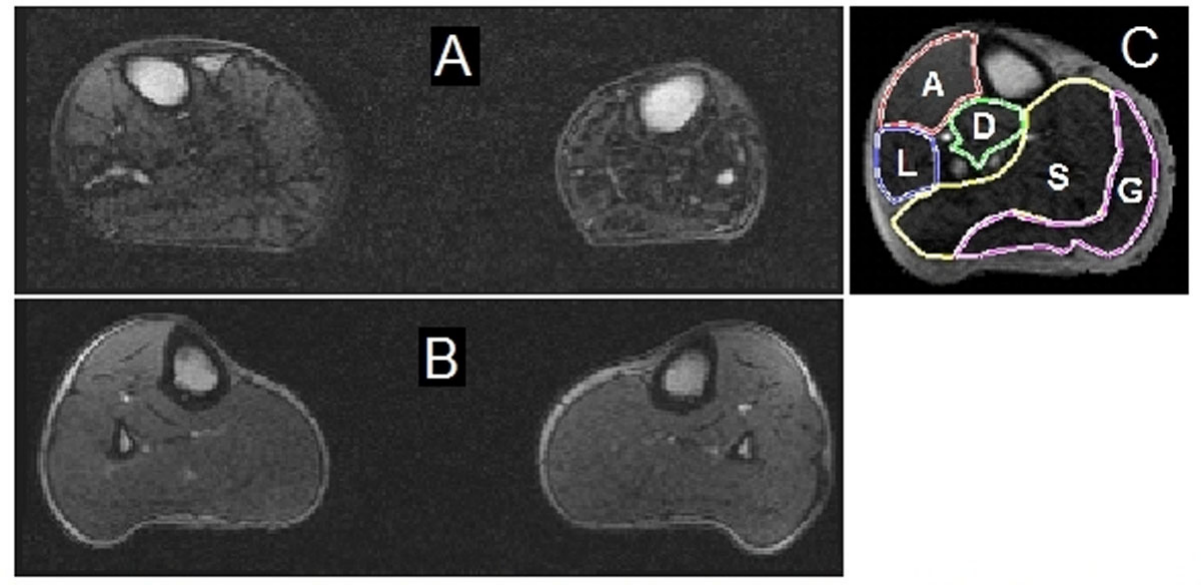
CE-MRI images from a PAD patient (panel A) and a healthy control (panel B) at the mid calf level obtained with a Siemens 3.0T Verio. Panal C shows the result of the semi-automatically segmented leg muscle groups. The contours show individual muscle compartments G: gastoccnemius; S: soleus; D: deep posterior muscle group; A: anterior muscle group; L: lateral muscle group.

## Conclusions

The preliminary data suggest that CE-MRI based texture analysis can quantify differences in leg muscle morphology between PAD patients and controls. Although the proposed texture analysis requires histological validation with leg muscle composition, muscle morphology might be of interest as a non-invasive imaging marker of PAD.

## Funding

This work was supported in part by The Methodist DeBakey Heart & Vascular Center (MDHVC) Research Award 2011, the Society for Vascular Surgery (SVS): 2012 Clinical Research Seed Grant, and NIH grant T32HL07812.

